# Trajectories through child protection and education: Patterns, timing and causality

**DOI:** 10.23889/ijpds.v1i1.317

**Published:** 2017-04-19

**Authors:** Miriam Maclean, Catherine Taylor, Melissa O'Donnell

**Affiliations:** University of Western Australia

## Objectives

1. To examine the reading trajectories of children with different levels of child protection involvement from Year 3-9 of school
2. To estimate the `effects' of entering care on Year 9 reading achievement, attendance and suspensions among children with substantiated maltreatment

## Approach

Record-linkage of population data was used to investigate educational outcomes for children born in Western Australia from 1990-2010. Data from the Departments of Health, Child Protection and Family Support, Education, and Disability Services were used. Multi-level modelling was used to assess children's reading trajectories from Year 3-9, and identify child, family, neighbourhood and child protection factors associated with reading achievement over time. Educational outcomes for maltreated children placed in out-of-home care were compared to a propensity-matched comparison group of children that remained at home using regression analyses.

## Results

Child protection involved children most often showed a stable pattern of low achievement from Year 3-9. Of those with mid-level Year 3 scores, 45%-50% showed declining achievement with scores in the lowest third of their Year 9 cohort. Particularly low achievement in Year 3 and 9 was found among children with early unsubstantiated maltreatment followed by older-aged entry to care. Propensity matched analysis showed that after controlling for maltreatment, child, family, and neighbourhood characteristics, maltreated children did not significantly differ by placement status on reading or suspensions. Absences were significantly lower among children that entered care versus those remaining at home (OR=0.36,95%CI[0.15, 0.91). 

## Conclusions

Findings suggest that poor educational outcomes for children that have entered care are not primarily caused by out-of-home care, but reflect prior disadvantage and maltreatment. Child protection involved children are more likely to show stable low and declining patterns of achievement than other children, highlighting a need not only for early intervention, but also for interventions to address academic problems that arise later in childhood.

